# Molecular Signatures of Prostate Stem Cells Reveal Novel Signaling Pathways and Provide Insights into Prostate Cancer

**DOI:** 10.1371/journal.pone.0005722

**Published:** 2009-05-29

**Authors:** Roy Blum, Rashmi Gupta, Patricia E. Burger, Christopher S. Ontiveros, Sarah N. Salm, Xiaozhong Xiong, Alexander Kamb, Holger Wesche, Lisa Marshall, Gene Cutler, Xiangyun Wang, Jiri Zavadil, David Moscatelli, E. Lynette Wilson

**Affiliations:** 1 Department of Cell Biology, New York University School of Medicine, New York, New York, United States of America; 2 Division of Immunology, University of Cape Town, Cape Town, South Africa; 3 Department of Science, Borough of Manhattan Community College, City University of New York, New York, New York, United States of America; 4 Department of Pathology, New York University School of Medicine, New York, New York, United States of America; 5 Department of Urology, New York University School of Medicine, New York, New York, United States of America; 6 Amgen Inc, San Francisco, California, United States of America; 7 NYU Cancer Institute, New York University School of Medicine, New York, New York, United States of America; 8 Center for Health Informatics and Bioinformatics, NYU Medical Center, New York, New York, United States of America; Roswell Park Cancer Institute, United States of America

## Abstract

**Background:**

The global gene expression profiles of adult and fetal murine prostate stem cells were determined to define common and unique regulators whose misexpression might play a role in the development of prostate cancer.

**Methodology/Principal Findings:**

A distinctive core of transcriptional regulators common to both fetal and adult primitive prostate cells was identified as well as molecules that are exclusive to each population. Elements common to fetal and adult prostate stem cells include expression profiles of Wnt, Shh and other pathways identified in stem cells of other organs, signatures of the aryl-hydrocarbon receptor, and up-regulation of components of the aldehyde dehydrogenase/retinoic acid receptor axis. There is also a significant lipid metabolism signature, marked by overexpression of lipid metabolizing enzymes and the presence of the binding motif for Srebp1. The fetal stem cell population, characterized by more rapid proliferation and self-renewal, expresses regulators of the cell cycle, such as E2f, Nfy, Tead2 and Ap2, at elevated levels, while adult stem cells show a signature in which TGF-β has a prominent role. Finally, comparison of the signatures of primitive prostate cells with previously described profiles of human prostate tumors identified stem cell molecules and pathways with deregulated expression in prostate tumors including chromatin modifiers and the oncogene, Erg.

**Conclusions/Significance:**

Our data indicate that adult prostate stem or progenitor cells may acquire characteristics of self-renewing primitive fetal prostate cells during oncogenesis and suggest that aberrant activation of components of prostate stem cell pathways may contribute to the development of prostate tumors.

## Introduction

It is likely that the aberrant proliferation of prostate stem cells (PSC) and/or their progenitors contributes to prostate pathology. We determined the gene expression signatures of fetal and adult PSC (FPSC and APSC) to gain insights into the signaling pathways that characterize these two normal stem cell (SC) populations and compared these profiles with those of prostate tumor cells. Delineating these regulatory pathways may provide insight into the mechanisms that convert quiescent adult prostate cells into a proliferating compartment that gives rise to benign prostatic hyperplasia and carcinoma thus permitting the targeting of specific pathways to treat these diseases.

We have shown that epithelial cells with SC features are concentrated in the proximal ductal region, adjacent to the urethra [1]–[3]. These features include quiescence, high proliferative potential and the ability of single cells to give rise to ductal structures that contain both basal and luminal cells [2]–[4]. We have previously isolated, based on the expression of Sca-1 [2], two populations of cells that are capable of regenerating prostatic tissue in an *in vivo* prostate reconstitution assay. The first population, stem cells, has considerable growth potential, does not require androgen for survival, expresses high levels of Sca-1 and resides in the proximal region of ducts. Almost all Sca-1^Hi^ cells also express ∝6 integrin, an antigen expressed on primitive prostate cells [2]–[4]. The second population, transit-amplifying cells, has more limited growth potential, expresses lower levels of Sca-1, requires androgen for survival and is found in all ductal regions [2], [3]. A third population, fetal prostate stem cells, exists in the urogenital sinus from which the prostate develops [5]. The inner layer of epithelial cells of the murine urogenital sinus starts invading the outer layer of mesenchyme to form the ducts of the prostate gland after E16. Prior to this event, the urogenital sinus epithelium (UGE) containing primitive fetal prostate cells can be isolated easily from the urogenital sinus.

In order to identify molecules and pathways that are active in primitive prostate populations we determined the transcriptional profiles of four populations of cells: (i) UGE, enriched in FPSC, (ii) Sca-1^Hi^, cells that express high levels of Sca-1, enriched in APSC [2], [6], (iii) Sca-1^Lo^, cells that express medium to low levels of Sca-1 and are enriched in transit-amplifying cells [2], and (iv) Sca-1^Neg^, cells with no Sca-1 expression, that represent the most mature population and have almost no regenerative potential [2]. To gain insight into the regulatory layers of transcriptional networks active in primitive prostate cells, we performed a computational screen of cis-regulatory promoter motifs [7] to reveal those that are significantly enriched among the PSC genes. We also identified functional gene categories that are enriched in the primitive cells.

The fetal and adult SC populations expressed numerous known SC-related genes. Our analysis revealed significant enrichment of several transcription factor (TF)-binding site motifs in the promoters of expressed genes. The data indicate that FPSC and APSC have unique and common transcriptional programs and identify a number of the key features that enable the maintenance and self-renewal of the undifferentiated state. A number of the stem cell-related genes we identify may also participate in the development of prostate tumors, indicating that these molecules may delineate a subset of tumors with a more primitive and possibly a more aggressive phenotype.

## Materials and Methods

### Cell preparation, antibodies and FACS analysis

#### Ethics statement

All animal care and procedures were performed in compliance with New York University institutional review board requirements.

The proximal region of prostatic ducts (i.e., the portion of the ducts nearest the urethra) of 6 week old C57BL/6 mice were digested and cells were examined for antigen expression (**Table S1**) [1], [2]. Sca-1-labelled cells were sorted by FACS using a DakoCyomation MoFlo sorter into 3 populations according to the mean fluorescence intensity (MFI) of Sca-1 expression [2]. Cells (10,000–50,000) with the highest MFI (25%; Sca-1^Hi^), medium/low MFI (40%; Sca-1^Lo^) and those lacking Sca-1 expression (25%; Sca-1^Neg^) were collected in TRIzol (Invitrogen). UGE was isolated from the urogenital sinus of 16-day-old C57BL/6 murine embryos [8] and added to TRIzol. The expression levels of Aldh were determined by FACS analysis after staining with the Aldefluor reagent kit (StemCell Technologies).

### Real-Time PCR

One µg of total RNA was reverse-transcribed at 52°C for 1 hour using the Thermoscript RT-PCR system (Invitrogen). 20 ng of resultant cDNA was used in a Q-PCR reaction using an iCycler (Biorad) and pre-designed TaqMan Gene Expression Assays (Applied Biosystems). Cycle threshold values from three separate RNA samples were averaged; amounts of target were interpolated from standard curves and normalized to hypoxanthine guanine phosphoribosyl transferase.

### RNA isolation and microarray hybridization

We established transcriptional profiles for UGE, Sca-1^Hi^, Sca-1^Lo^, and for the Sca-1^Neg^ differentiated cells. Three replicates of UGE (FPSC) and Sca-1^Hi^ (APSC) samples and four replicates of Sca-1^Lo^ and Sca-1^Neg^ samples were analyzed. RNA was isolated by standard procedures and its quality was assessed using an Agilent 2100 Bioanalyzer (Agilent Technologies). Samples with a RNA Integrity Number (RIN) >7.0 were considered suitable for labeling and 20 ng were labeled using the GeneChip two-cycle target labeling kit (Affymetrix). Ten micrograms of labeled and fragmented cRNA were then hybridized to the mouse genome MOE430 2.0 array (Affymetrix) which interrogates ∼45,000 transcripts. Raw expression data (CEL files) were generated using GCOS 1.4 (Affymetrix). The data discussed in this publication have been deposited in NCBI's Gene Expression Omnibus and are accessible through GEO Series accession number GSE15580 (http://www.ncbi.nlm.nih.gov/geo/query/acc.cgi?acc=GSE15580).

### Analysis of gene expression data

Utilizing ArrayAssist (Stratagene), raw Affymetrix CEL files were processed by applying the MAS5 algorithm, to assign detection calls (Present/Marginal/Absent) for each probe set that were subsequently used in downstream data filtering. To generate normalized expression levels, we used PLIER (probe logarithmic intensity error) algorithm, a model-based, multi-array signal estimator which produces more accurate probe-set signal values [9]. The combination of these above metrics was used for data filtering to obtain 33,967 “*valid genes*”, representing transcripts (gene probes) with signals >20 and detected as Present in at least one sample. To obtain a subset of variable genes, we calculated the coefficient of variation (CV) for each transcript and generated a set of 5095 “*active genes*” containing the transcripts with the highest (15% of the total) CV scores. To focus on the genes that were altered in a statistically significant manner, the samples were grouped according to cell type (UGE, Sca-1^Hi^, Sca-1^Lo^, Sca-1^Neg^), intensities of the *active gene* transcripts were log2-transformed and subjected to further statistical analyses utilizing two different tests: (a) one-way ANOVA using a Benjamini-Hochberg correction (*p*<0.05) and (b) significance analysis of microarrays (SAM) method with a false discovery rate of 5%. This yielded a list of 3137 “*significant genes*”, representing transcripts that matched the criteria of both these statistical tests. A principle components analysis (PCA) (mean centered) calculated by ArrayAssist, showed an appropriate reproducibility and separation within the different types of cells (**Figure S1**). To define the distinctive characteristics of the UGE, Sca-1^Hi^ and Sca-1^Lo^ populations, we compared the gene expression intensity reads (grouped samples) of each of these populations with that of the most differentiated adult cells (Sca-1^Neg^) using an unpaired T-test (Benjamini-Hochberg corrected *p*<0.05). Three gene subsets (UGE, Sca-1^Hi^ and Sca-1^Lo^) that contain transcripts whose expression increased (>1.75-fold) relative to their expression in Sca-1^Neg^ cells were generated.

### Analysis of functional categories

We utilized functional annotations of murine genes provided by the Murine Genome Informatics, which uses the standard vocabulary introduced by the Gene Ontology (GO) consortium. Enriched functional categories (*p*≤0.01, after correction for multiple testing) were identified in each of the three clusters (*UGE-only*, *UGE+Sca-1^Hi^*, *Sca-1^Hi^-only*) using EXPANDER, in which hypergeometric calculation is used to determine over-represented GO functional categories in a target set relative to a background set (the entire collection of putative murine genes) [7]. To avoid biases, genes represented by multiple probe sets were counted only once.

### Computational analysis of promoter cis-regulatory elements

For promoter analysis we applied EXPANDER [7] to detect cis-regulatory promoter elements that control the observed transcriptional alterations in the gene expression clusters. Given target and background sets of promoters, EXPANDER performs statistical tests to identify TFs whose binding-site signatures are significantly over-represented in the target set relative to background (TF enrichment is indicated by *p*-value) [7]. Both strands of each promoter were scanned for putative binding sites (spanning the transcriptional start site from 1000 bp upstream to 200 bp downstream). The enrichments identified in this study were robust, as they remained stable over a large range of threshold values.

### Comparison of data to known SC-profiles

Entrez Gene IDs and UniGene unique identifiers were used to match genes represented in different microarray platforms. We scored the number of genes that were commonly up-regulated in the gene expression profiles and in at least one other SC profile.

## Results and Discussion

### Profiling of gene expression in three prostate stem/progenitor cell enriched populations

**Figure 1:**
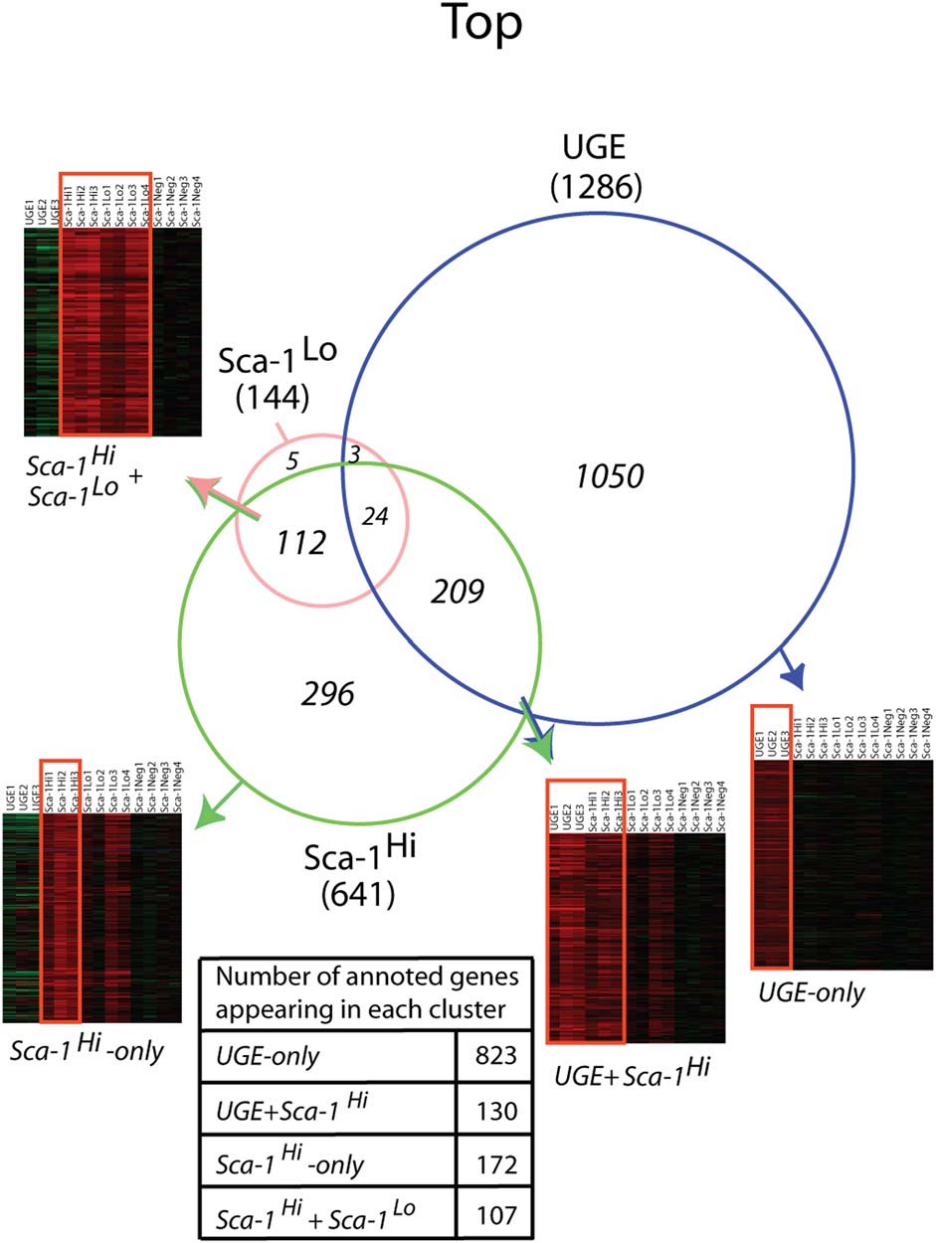
Overlapping gene expression in primitive prostate cell populations. A Venn diagram detailing the number of transcripts (gene probes) of overexpressed genes shared and distinct among UGE, Sca-1^Hi^ and Sca-1^Lo^ subsets. The number of transcripts within each subset is given in brackets outside the Venn chart. The number of transcripts within each cluster is given in Italics inside the slices of Venn chart. Numbers of annotated genes appearing in each of the four clusters are given in the inset table. The corresponding dendogram of each cluster is presented.

RNA was isolated from the four cell populations described above (UGE, Sca-1^Hi^, Sca-1^Lo^ and Sca-1^Neg^), prepared for hybridization to microarrays and analyzed as described in Materials and Methods. Three gene subsets (UGE, Sca-1^Hi^, Sca-1^Lo^) were generated consisting of transcripts whose expression was statistically elevated in each of the populations relative to their expression in the most differentiated Sca-1^Neg^ cell population (**Figure 1** **,** **Table S2**). The FPSC subset has the highest number of significantly altered transcripts (1286 gene probes), as may be expected when comparing a fetal SC with a differentiated adult cell population. The APSC subset (Sca-1^Hi^) has 641 and the transit-amplifying subset (Sca-1^Lo^; more differentiated than the APSC subset) has only 144 transcripts that are differentially expressed. We then determined whether we could discern common expression patterns among these three progenitor subsets and patterns that were unique to each subset. Intersection of the up-regulated transcripts within the three subsets resulted in the generation of four clusters (*UGE-only*, *UGE+Sca-1^Hi^*, *Sca-1^Hi^-only*, *Sca-1^Hi^*+*Sca-1^Lo^*) of mutually or exclusively expressed genes (**Figure 1** **;** **Table S3**). The most distinctive gene expression pattern was derived from the UGE cell population, manifested by 1050 transcripts that were exclusively overexpressed in the *UGE-only* cluster (FPSC cluster). A second distinctive pattern was evident in the Sca-1^Hi^ subset, represented by the exclusive up-regulation of 296 transcripts in the *Sca-1^Hi^-only* cluster (APSC cluster). Interestingly, within the cluster that overlaps between the fetal and the adult subsets, the *UGE+Sca-1^Hi^* cluster, we found substantial numbers of transcripts (209) that were commonly up-regulated, while lesser commonality (112 transcripts) was observed in the cluster that overlaps (*Sca-1^Hi^*+*Sca-1^Lo^* cluster) between the APSC subset (Sca-1^Hi^) and the TA subset (Sca-1^Lo^) (**Figure 1** **,** **Table S2**). Very few transcripts (5) were distinctively overexpressed in the more differentiated population (*Sca-1^Lo^-only* cluster). These results depict prominent stage-specific gene transcripts expressed during the maturation of prostate cells, starting from the most primitive fetal population (UGE), and progressing through the APSC population (Sca-1^Hi^), and its progeny, the transit-amplifying cells (Sca-1^Lo^), and culminating with the most differentiated subset (Sca-1^Neg^).

### A. Expression of SC markers in prostatic stem/progenitor clusters

**Figure 2:**
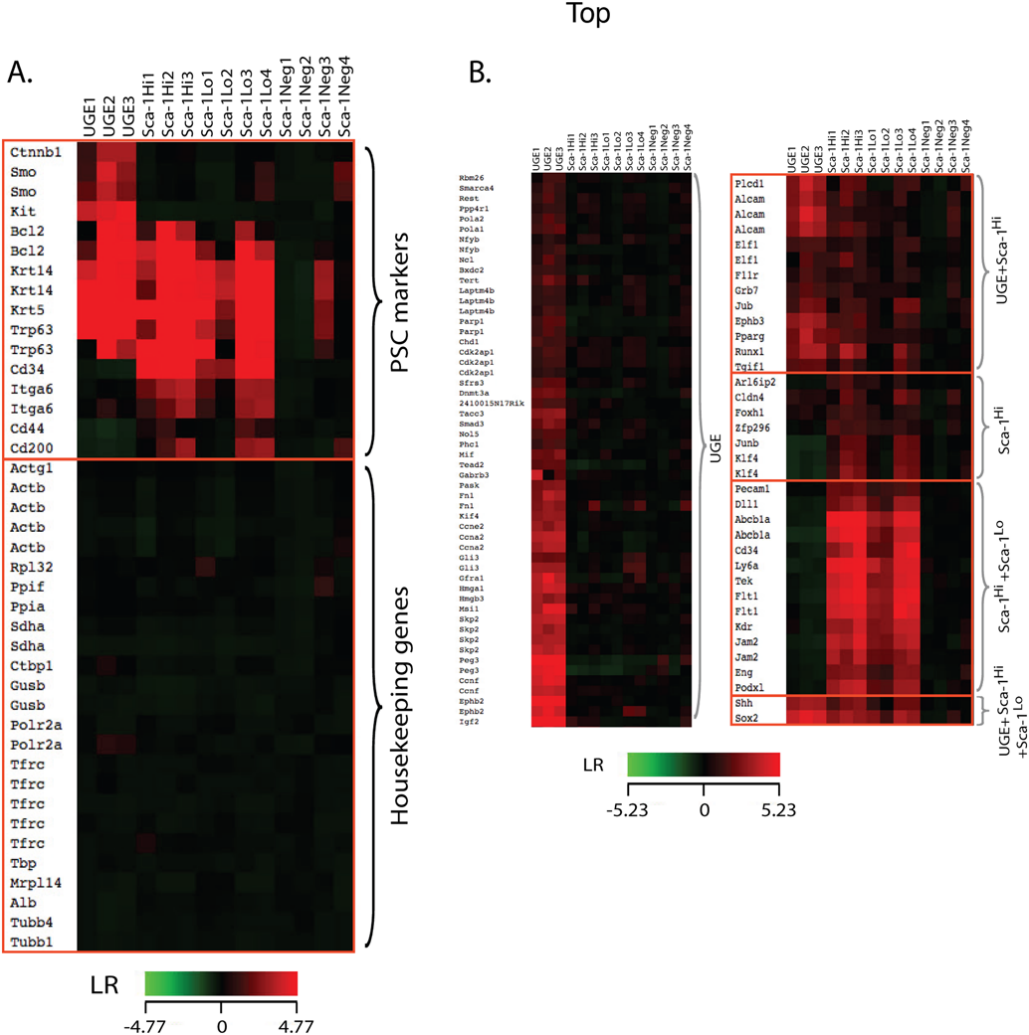
Known markers of primitive prostate (A) and stem (B) cells are expressed. A. Expression profiles of molecules described as being expressed in primitive prostate populations that were up-regulated at least 2-fold (upper panel) in the UGE, Sca-1^Hi^ and Sca-1^Lo^ cells are presented. Each column represents an individual sample, and each row represents a specific gene. Red (high), green (low) relative expression; black indicates equal expression relative to the Sca-1^Neg^ cells. The lower panel presents a cassette of 15 common murine housekeeping genes that manifest stable expression across all samples. Log ratio (LR) values are presented in **Table S3**. B. Expression profiles of known SC markers (*eGOn* and *FatiGO* survey), that were up-regulated by at least 2-fold in prostate stem/progenitor enriched samples. Five expression patterns can be distinguished. LR values are presented in **Table S5**.

**Figure 3:**
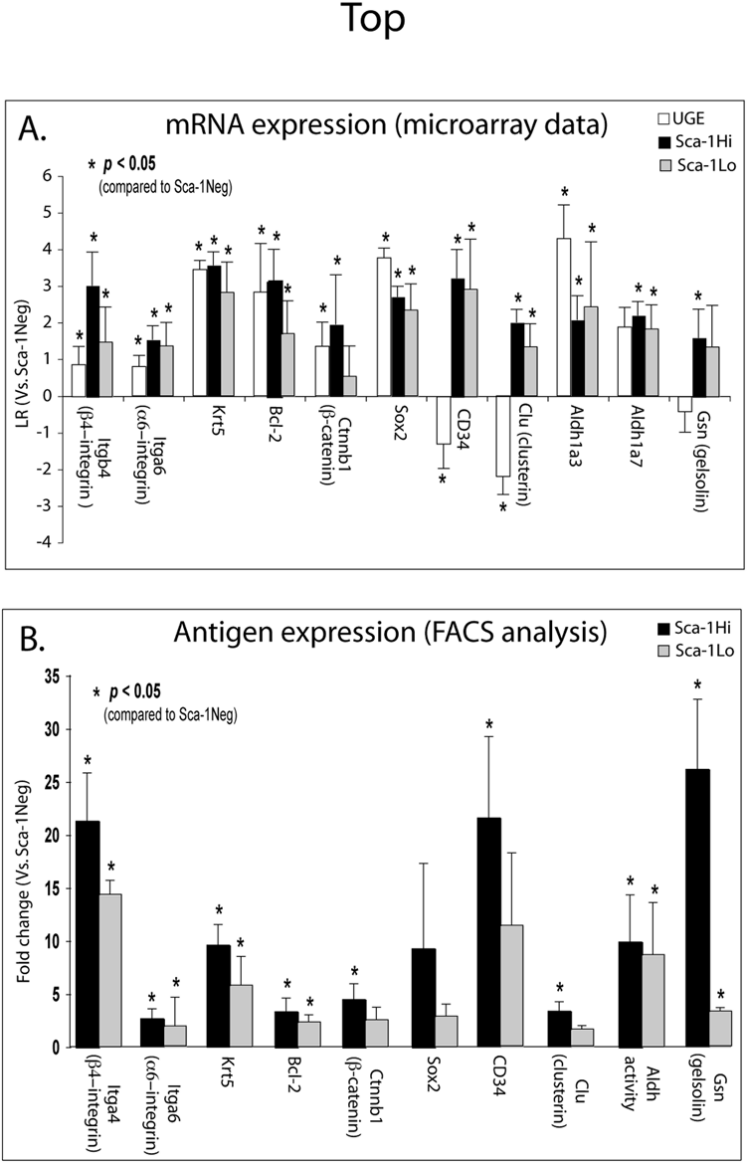
Transcript and protein expression of SC markers in primitive (UGE, Sca-1^Hi^) and progenitor (Sca-1^Lo^) cells. A. Transcription level of SC markers in primitive and progenitor prostate cells. Microarray data of SC markers from these primitive cell populations are expressed as LR values [mean±SD] relative to mature Sca-1^Neg^ samples. B. Antigen expression of SC markers on Sca-1^Hi^ and Sca-1^Lo^ cells. FACS analysis of Sca-1^Hi^ and Sca-1^Lo^ cells determined the expression of SC markers (denoted in A) on these populations. Enrichment values [mean±SD] are expressed as the fold change in antigen expression relative to the antigen expression of the mature Sca-1^Neg^ cell population.

Our profiling analysis indicates that all three stem/progenitor enriched subsets contain substantial numbers of known markers of murine PSC, namely *Trp63*, *Cd200*, *Ctnnb1*, *Smo*, *Krt5*, *Krt14*, *Itga6*/*Cd49f*, *Cd44*, *Kit*, *Bcl2*, and *Cd34* [10] (**Figure 2A, B** **,** **Table S4**). A comparison of 11 known PSC markers that were up-regulated in our subsets with a panel of 15 common murine housekeeping genes, whose expression was not altered, confirms that our profiles reflect a specific transcriptional signature of primitive FPSC and APSC (**Figure 2A** **;** **Table S4**). In addition, transcripts for an ephrin receptor, *Ephb3*, and two ephrin ligands, *Efna5* and *Efnb2*, which act as coordinators of migration and proliferation in the intestinal SC niche [11] are up-regulated in both the FPSC and APSC populations (**Figure 2B**). FACS analysis of Sca-1^Hi^ and Sca-1^Lo^ cells validated the increased expression of several stem cell antigens predicted by the microarray (**Figure 3A**) including stem cell markers α6 and β4 –integrins [12], keratin 5, Bcl-2, β-catenin [10], Sox2 [13] and CD34 [14] (**Figure 3B**). This indicates that changes in mRNA levels of many SC-markers are reflected by changes in their protein levels.

To determine if additional SC related genes were expressed in the prostate stem/progenitor subsets, we used two approaches. First, we utilized two bioinformatics tools (*eGOn* and *FatiGO* [15]) for identifying genes annotated as stem cell-genes based on prior publications. This survey indicates that all our stem/progenitor-related subsets (UGE, Sca-1^Hi^, Sca-1^Lo^) express a number of stem cell markers (total of 67 genes) (**Figure 2B** **;** **Table S5**). Our second approach for determining the primitive nature of the profiles involved a systematic comparison of our profile with five other gene expression profiles from embryonic (ESC), hematopoietic (HSC), neuronal (NSC) [16], [17], skin [18] and liver [19] stem cells. We found that significant numbers of mRNAs expressed in the prostate stem/progenitor clusters were also up-regulated in at least one of the five published SC profiles (ranging from 40% of the *UGE-only* mRNAs to 20% of the *Sca-1^Hi^+Sca-1^Lo^* mRNAs) (**Figure 2B** **,** **Tables 1** **,** **S6**). Thus, despite the limitations of comparing data obtained using different experimental conditions and less comprehensive microarrays than used in our study, we detected a high degree of overlap between genes overexpressed in the prostate stem/progenitor profiles and those that are overexpressed in other SC profiles. This implies that prostate stem/progenitor cells share numerous features with SC isolated from other sources. However, prostate stem/progenitor cells also express genes not identified in any of these five SC populations indicating that some of these genes may be unique to prostate or may be shared with other undescribed SC profiles. Notably, the two approaches used for estimation of SC-marker enrichment indicate that while there is considerable overrepresentation of SC-related genes in adult Sca-1^Hi^ cells, there is an even higher representation of SC-markers in UGE cells. Interestingly, a comparison with two ‘stemness signature’ studies [16], [17] indicates that the PSC profile has greater overlap with ESC or NSC profiles, than with HSC. For example, comparison with the data of Ramalho-Santos et al [17] indicates that 14.4% and 16.1% of the PSC-enriched mRNAs overlap with the ESC- and NSC-enriched mRNAs respectively, whereas fewer mRNAs (6.8%) overlap with HSC-enriched transcripts (**Table 1**). Several recent studies have shown that gene expression profiles of ESCs and NSCs overlap with each other to a greater extent than with HSC [17], [20]. Accordingly, our results suggest that the prostate progenitor lineage may resemble that of neuronal or embryonal progenitors to a greater extent than that of hematopoietic progenitors.

**Table 1:** Comparison of primitive prostate profiles with other SC profiles.

**Ivanova et al.**	**ESC (2270)**	**(%)**	**HSC (2359)**	**(%)**	**NSC (2492)**	**(%)**	**Number of gene probes that are in common with at least one other profile (ESC,HSC,NSC)**	**(%)**			
*UGE-only (1050)*	117	**11.1**	77	**7.3**	106	**10.1**	201	**19.1**			
*UGE+SCA1-Hi (209)*	21	**10.0**	13	**6.2**	22	**10.5**	40	**19.1**			
*SCA1-Hi-only (296)*	8	**2.7**	21	**7.1**	13	**4.4**	35	**11.8**			
*SCA1-Hi+SCA-Lo (112)*	3	**2.7**	12	**10.7**	7	**6.3**	17	**15.2**			
*All sections (1667)*	149	**8.9**	123	**7.4**	148	**8.9**	293	**17.6**			
**Ramalho-Santos et al.**	**ESC (1787)**	**(%)**	**HSC (1977)**	**(%)**	**NSC (2458)**	**(%)**	**Number of gene probes that are in common with at least one other profile (ESC,HSC,NSC)**	**(%)**			
*UGE-only (1050)*	203	**19.3**	64	**6.1**	242	**23.0**	325	**31.0**			
*UGE+SCA1-Hi (209)*	10	**4.8**	6	**2.9**	3	**1.4**	16	**7.7**			
*SCA1-Hi-only (296)*	23	**7.8**	36	**12.2**	20	**6.8**	59	**19.9**			
*SCA1-Hi+SCA-Lo (112)*	4	**3.6**	7	**6.3**	3	**2.7**	12	**10.7**			
*All sections (1667)*	240	**14.4**	113	**6.8**	268	**16.1**	412	**24.7**			
**Tumbar et al.**	**Skin SC (152)**	**(%)**									
*UGE-only (1050)*	18	**1.7**									
*UGE+SCA1-Hi (209)*	3	**1.4**									
*SCA1-Hi-only (296)*	2	**0.7**									
*SCA1-Hi+SCA-Lo (112)*	0	**0.0**									
*All sections (1667)*	23	**1.4**									
**Petkov et al.**	**Hepatic SC (282)**	**(%)**							**Summary Table**	**Number of gene probes that are in common with at least one other profile**	**(%)**
*UGE-only (1050)*	24	**2.3**							*UGE-only (1050)*	425	**40.5**
*UGE+SCA1-Hi (209)*	1	**0.5**							*UGE+SCA1-Hi (209)*	51	**24.4**
*SCA1-Hi-only (296)*	4	**1.4**							*SCA1-Hi-only (296)*	76	**25.7**
*SCA1-Hi+SCA-Lo (112)*	0	**0.0**							*SCA1-Hi+SCA-Lo (112)*	22	**19.6**
*All sections (1667)*	29	**1.7**							*All sections (1667)*	574	**34.4**

A summary of the number of transcripts expressed in each of the isolated prostate cell clusters in comparison with defined SC profiles from ESC, HSC and NSC [16], [17], skin SC [18] and liver SC [19]. The number of transcripts appearing in the prostate cluster or SC publication is denoted in brackets in each case. In each instance the percentage (%) indicates the correspondence with the prostate cluster. A complete list of transcripts for each of the denoted comparisons is presented in Table S5.

The presence of significant numbers of SC genes in our APSC and FPSC suggests that these populations have the characteristics of undifferentiated progenitor cells. We next determined the molecular profiles of the isolated PSC populations to decipher potential signaling pathways that are expressed by these cells.

### B. Identification of overrepresented functional categories and TF-binding promoter motifs within the clusters of genes that are overexpressed in the stem/progenitor cells

To identify the major biological processes and transcriptional networks within the three SC-containing clusters (*UGE-only*, *UGE+Sca-1^Hi^*, *Sca-1^Hi^-only*; **Figure 1**) we applied the EXPANDER package for functional and promoter analysis. A number of functional categories and TF-binding promoter motifs are significantly enriched in these clusters (**Tables 2** **,** **S7**).

**Table 2:** Promoter analysis of primitive prostate clusters.

Cluster	TF	Accession number in TRANSFAC DB	p-value*	Enrichment Factor**
***UGE-only (FPSC)***	E2f	M00918	4.7E-14	1.92
	Nfy	M00287	4.9E-14	1.63
	ETF/Tead2	M00695	1.2E-09	1.44
	Ap2	M00189	1.5E-05	1.29
	AhR/Arnt	M00235	1.1E-03	1.32
***UGE+Sca-1Hi (FPSC and APSC)***	Srebp1	M00749	5.5E-04	1.66
	Ap2	M00189	4.9E-03	1.31
	Smad3	M00701	5.2E-03	1.76
	Fxr/Rxra	M00631	7.3E-03	2.00
***Sca-1Hi-only (APSC)***	Smad	M00974	2.8E-03	1.80
	Srebp1	M00221	8.6E-03	1.59
	T3r (Rxr-a or b)	M00963	1.1E-02	1.73
	Smad3	M00701	2.2E-02	1.54

TFs whose binding site profiles were significantly enriched in the three SC-related clusters described in Fig. 1 are presented.

* P values indicate the significance of TF signature enrichment in the cluster relative to that in the background set as described in Materials and Methods.

** Enrichment factor values represent the frequency of the TF signature in a cluster divided by its frequency in the background set.

### a) Self-renewal signature in FPSC

The *UGE-only* cluster is highly enriched in cell proliferation genes , as would be expected for an expanding fetal population (**Tables 3** **,** **S7** **,** **8**). Up-regulation of *Mki67* (12-fold) exclusively in the UGE subset attests that these cells are proliferating. Proliferation and cell cycle-related pathways are elevated in the self-renewal of normal SC [21]. PTEN deletion, which enlarges the pool of self-renewing NSC [21], reveals a SC-self-renewal signature (231 genes) for these cells. Notably, approximately 64% of these genes are also up-regulated in the *UGE-only* cluster, indicating considerable similarity in gene expression between these two self-renewing populations (**Table S9**). Components, as well as target genes, of the Wnt/β-catenin pathway that promotes self-renewal in many types of SC [22] are strongly represented in FPSC and APSC (**Tables 3** **,** **S10**). The aberrant activation of this pathway in prostate tumors [23] and its enrichment in PSC is consistent with the notion that prostate cancer may arise in the primitive compartment. qPCR analysis validated the gene expression profiles, indicating that expression of *Wnt4* is elevated in FPSC (5-fold ) and APSC (8-fold) relative to mature cells (Sca-1^Neg^). In addition, *Wnt6* is elevated in FPSC (22-fold), and Fzd6 is elevated in FPSC (5-fold) and APSC (3-fold) relative to mature cells (**Table S11**). Components of the sonic hedgehog (Shh) pathway, which is also implicated in the self-renewal of primitive cells [24] and the suppression of differentiation, are manifest in FPSC and APSC (**Tables 3** **,** **S12**). The abundance of Shh-regulators and target genes that are up-regulated in the FPSC and APSC indicates that autocrine hedgehog signaling may play an important role in prostate progenitor/stem cell biology. qPCR analysis validates the gene expression data and indicates that *Shh* and *Gli3* expression are increased in fetal (35-fold and 4-fold respectively) and adult (6-fold and 3-fold respectively) SC relative to mature cells (**Table S11**). Hedgehog signaling is important for normal prostate growth and increases during prostate tumorigenesis in concert with an increase in progenitor cell markers [25], implying again that primitive cells may be expanded during tumorigenesis.

**Table 3:** Transcriptional profile of genes expressed in (i) UGE, (ii) in both UGE and Sca-1Hi and (iii) Sca-1Hi subsets relative to the Sca-1Neg subset.

Subset	Category	mRNAs
**UGE (FPSC)**	Cell cycle	**Cdc2a (x10)** *, **E2f3 (x3)**, **Tead2 (x4)**, Cdc25c (x4), Cdkn3 (x3), Cdk4 (x7), Mcm2 (x5), Mcm6 (x7), Nras (x5), Ccna2 (x9) , Ccne1 (x53), Brca1 (x4), Mki67 (x12)
	Chromatin modifiers	**Ezh2 (x9)**, Hdac2 (x3), Bmi1 (x2), Jarid1b (x5), Hmga1 (x11), Hmga2 (x47), Suv39h2 (x4), Dnmt3a (x4)
	Notch signaling	Lfng (x4), Dll1 (x2) Sdcbp2 (x3)
	E2f-target genes	**Cdc2a (x10)**, Cdc6 (x7), Ccne1 (x52), Dhfr (x5), Mcm2 (x4), Mcm5 (x8), Mcm6 (x6), Orc1l (x9), Tk1 (x4), Tyms (x5), Ccna2 (x9)**, Cdca3 (x6)**, Igf2 (x28)**, Mad2l1 (x5)**, Mal2 (x10)**, Rrm2 (x4)**, Tpx2 (x10)**, Ube2c (x5)**, Plk1 (x9)**
	Nfy-target genes	**Ccnb1 (x12)**, Ccnb2 (x8), Plk1 (x9), Cdc25c (x2), Ccna2 (x9), Col11a1 (x5), Cdc2a (x10), Hes1 (x2), Tert (x3)
	Ap2-target genes	Igf2 (x27), Lor (x28), Cdh1 (x3), Erbb2 (x2)
**UGE and Sca-1Hi (FPSC and APSC)**	Shh signaling	**Shh (x7, x4)**, **Gli3 (x7,NC)**, Mycn (x82, x2), Hhat (x2, NC), Disp1 (x2, NC), Sufu (x2, NC), Smo (x3, NC), Chek (x8, NC), Ptch1 (x2, NC)
	Wnt Signaling	**Wnt4 (x9, x5)**, **Wnt6 (x8, NC)**, **Fzd6 (x8, x3)**, **Ctnnb1 (x2, NC)**, Wnt7a (x37, NC), Csnk1g1 (x2, NC), Fzd2 (x3, x-2), Fzd3 (x2, NC), Wnt7b (x4, x2), Wnt9b (x4, NC), Wnt10a (x14, x4), Lrp6 (x2, x3)
	Ahr pathway and target genes	**Ahr (x4, x5)**, Tk1 (x5, NC), Syt9 (x17, NC), Cd1d1 (x9, NC), Arnt2 (x5, NC), Smad3 (x5, NC), Magi3 (x4, NC), S100g (x4, NC), Snn (x6, NC), Col11a1 (x5, NC), Mycl1 (x5, NC), Slc2a1 (x5, NC), Lamb3 (x3, x6), Zfp36l1 (x2, x2), Sprr1a (x21, x21), Cdh3 (x5, x2)
	ALDH/RA/Retinoid recepror axis	**Aldh1a2 (x4, x -7)** ***, **Rxra (x4, NC)**, **Rarg (x2, x3)**, **Rarb (x6, NC)**, **Crabp2 (x15, NC)**, Aldh1a3 (x7, x2), Aldh1a7 (x4, x4), Aldh3a1 (x -7, x6), Aldh3b2 (x4, NC)
	Epithelial morphogenesis factors	Krt4 (x28, x28), Krt6a (x16, x2), Alcam (x5, x2), Efna5 (x9, x2), Efnb2 (x9, x3), Ephb3 (x7, x3), Celsr1 (x18, x4), Evpl (x10, x4), Ppl (x11, x6)
	Phospholipid metabolism	**Rxra (x4, NC)**, **Pltp (x2, x8)**, Akp2 (x2, x2), Lypd2 (x23, x3), Lypd3 (x25, x2), Anxa8 (x8, x6), Lgals3 (x3, x8), Dlk1 (x9, x2), Ly6a (x-2, x18), Tiam1 (x12, x3), Fasn (x2, NC), Hmgcr (x4, NC)
	TGFb-pathway and target genes	**Clu (x-4, x4)**, Mmp11 (x8, NC), Smad2 (x2, NC), Smad3 (x3, NC), Lrp1 (x3, x5), Klf4 (x-4, x4), Eng (NC, x4), Junb (x-5, x3), Acvrl1 (NC, x3), Rhoa (NC, x4), Itgfbp3 (NC, x14), Itgb6 (NC, x3)
	Detoxification and protection from oxidative stress	**Ahr (x4, x5)**, Aldh1a2 (x4, x -7), Aldh1a3 (x7, x2), Aldh1a7 (x4, x4), Ahr (x4, x5), Cyp2s1 (x2, x4), Cyp2c29 (x2, x3), Cyp51 (x2, NC), Cyp4f39 (x37, NC), Cyp26b1 (NC, x2)
	ABC transporters	Abcb1a (x-3, x18 ), Abcd3 (NC, x3), Abcc3 (NC, x3), Abcc4 (x4, x4 ), Abcc5 (NC, x2)
**Sca-1Hi (APSC)**	Calcium dependent regulators	**Thbd (x12)**, **Itpr3 (x2)**, Anxa3 (x6), S100s10 (x22), Cacna2d4 (x3), Trpc2 (x2), Cbara1 (x6), Ryr2 (-5), Ryr3 (x-3), Itpr2 (x2),

Functional classification is shown for 146 mRNAs scored as increased relative to differentiated Sca-1Neg cells (Table S3). The average relative increases (fold change) from three comparisons is given in parentheses. When two values are presented in brackets following a gene, the first number represents the fold change in UGE, the second in Sca-1Hi. A mRNA that is present but not increased is denoted NC.

* Bolded genes represent those that were validated by FACS or qPCR.

** Specific E2f3-target genes.

*** Aldh activity is elevated in Sca-1Hi cells (Figure 3B).

TF-binding site analysis of promoters for genes that were up-regulated within the FPSC (the *UGE-only* cluster) identifies two transcriptional regulators of the cell cycle, namely E2f and Nfy (**Tables 2** **,** **S13**), consistent with over-representation of self-renewal genes (**Tables 3** **,** **S7**) [20]. Importantly, increases in the levels of mRNAs encoding four members of the E2f family are observed along with numerous genes that are specific targets of E2f3 (**Table 3**). Analysis by qPCR confirms that *E2f3* (3-fold) and its representative target gene, *Cdc2a* (12-fold), are up-regulated in the UGE cells compared with mature cells (**Table S11**). Interestingly, E2f3 expression is associated with the self-renewal of murine trophoblast SC [26], the expansion of SC in the columella and lateral root caps of Arabidopsis [27], and a poor prognosis in prostate cancer [28]. The binding-motif signature for Nfy is also well represented in the promoters of FPSC genes and is in accordance with the increased transcription of Nfya and Nfyb subunits and the finding that Nfya is a potent inducer of HSC self-renewal [29].

Additional promoter signatures that are enriched in the *UGE-only* cluster are those of Ap2 and of the embryonic TEA domain-containing factor (ETF), also known as Tead2 (**Tables 2** **,** **S13**). In the FPSC expression of *ETF*/*Tead2* and its coactivator, *Yap1*, were increased by 4- and 2- fold respectively. The increased expression of *Tead2* (4-fold) in FPSC relative to mature cells was verified by qPCR (**Table S11**). Tead2 induces genes that promote the self-renewal of progenitor cells in the olfactory epithelium [30] and is essential during murine embryo development [31]. Ap2 transcripts are elevated 3-fold and a number of its target genes are increased (**Table 3**). Ap2 promotes proliferation over differentiation and is a marker of SC and pluripotency [32]. Thus, E2f, Nfy, Tead2 and Ap2 are likely to be involved in the self-renewal and expansion of the primitive prostate population.

### b) TGF-ß signaling signature in APSC

In contrast to the abundance of proliferation genes present in the expanding UGE population, in APSC (Sca-1^Hi^ subset) we find that TGF-β target genes are upregulated indicating that TGF-β signaling is a prominent feature of APSC. We previously documented that the maintenance of dormancy of APSC is dependent on the TGF-β/Smad2/3 signaling pathway [33]. Significantly, our cis-element promoter analysis identifies enriched binding-sites for Smad and Smad3 in the *Sca-1^Hi^-only* cluster, while similar sites were absent from the *UGE-only* cluster (**Table 2**), indicating that TGF-β/Smad signaling may have a predominant role in APSC.

The expression level of *Itgb6*, an integrin that binds and activates TGF-β [34] is up-regulated 3-fold in APSC (**Table 3**). In addition, an increase in the expression levels of *Igfbp3* (14-fold), that phosphorylates Smad3 [35], is observed exclusively in the Sca-1^Hi^ subset. Validation by qPCR (3-fold; **Table S11**) and FACS analysis (3-fold; **Figure 3B**) confirms that the TGF-β target gene clusterin (Clu) is up-regulated in APSC. Our promoter analysis also identifies overrepresentation of the SMAD3 motif in the promoters of genes from the UGE+Sca-1^Hi^ cluster (**Table 2**), indicating that TGF-β may also mediate signaling in the FPSC. As a second approach for determining the possible involvement of TGF-β signaling in primitive prostate cells, we compared the genes from the three SC-enriched clusters (*UGE-only*, *UGE+Sca-1^Hi^* and *Sca-1^Hi^–only*) with the TGF-β-driven signature from keratinocytes [36]. These comparisons indicate that approximately 14% of the genes in each of these three tested clusters may be TGF-β targets (*UGE-only* 112/823; *UGE+Sca-1^Hi^* 19/130; *Sca-1^Hi^–only* 23/172 genes) (**Table S14**), supporting the contribution of TGF-β signaling in FPSC in addition to its prominent role in the APSC (**Table 3**). In this regard it is important to note that *Smad2* and *Smad3*, the two main transcriptional mediators of TGF-β, are exclusively up-regulated in the UGE (**Table 3**). The prominent representation of the Smad binding-site motif in the promoters of genes that are up-regulated in the APSC (Sca-1^Hi^-only cluster) indicates that the TGF-β signaling pathway is highly relevant to the transcriptional program of these cells and supports the finding that TGF-β maintains the quiescence of APSC [33]. In FPSC the inhibitory effects of TGF-β may be negated by the expression of numerous molecules related to self-renewal (see above). It is also possible that TGF-β may have a different function in primitive fetal prostate cells and that it may promote their self-renewal and maintenance as has been shown in embryonic cells [37]. In summary, we find that FPSC express many regulators that participate in self-renewal as expected in a developing fetal organ. In contrast, target genes of the TGF-β signaling pathway, which maintains dormancy in the adult prostate stem cell niche [33] are significant features of APSC.

### c) The Ahr/Arnt pathway is expressed in fetal and adult PSC

The aryl-hydrocarbon receptor (Ahr)/Ahr receptor nuclear translocator (Arnt) pathway has important roles in organ development and dioxin toxicology and may be essential for protection against oxidative stress [38]. As the importance of Ahr in prostate biology is well documented and its deficiency results in a smaller prostate [39] and in increased susceptibility to prostate tumors [40], it is of interest that the binding site of this TF is overrepresented in the promoters of genes of the *UGE-only* cluster (**Table 2**). Microarray data (**Tables 3** **,** **S13**) and qPCR analysis (FPSC 2-fold; APSC 3-fold; **Table S11**) both indicate that *Ahr* and many of its target genes are up-regulated in FPSC and APSC, implying that the Ahr/Arnt complex is transcriptionally active in PSC. Exposure of mice to the Ahr agonist, dioxin, increases the absolute number of bone marrow SC [41], but, surprisingly, these cells are deficient in self-renewal and have impaired regenerative potential [42]. Similar to its effects on HSC, dioxin also inhibits fetal prostatic bud formation [43]. As Ahr/Arnt promoter binding sites are enriched in the genes of the UGE subset, we propose that Ahr is necessary for the regulation of PSC activity and that its over activation may impair self-renewal and the subsequent expansion and differentiation of prostate progenitor cells.

### d) The Aldh/RA/retinoid receptor axis is expressed in PSC

An interesting category of genes up-regulated in the *UGE-only* cluster is that related to embryonic development (**Table S7**). This includes two aldehyde dehydrogenase (Aldh) enzymes, *Aldh1a2* and *Aldh1a3* (**Table S8**), which convert retinal into retinoic acid (RA) [44], a critical factor promoting differentiation. In addition, expression of *Crabp2* that shuttles RA into the nucleus to bind RA receptors [45], is exclusively up-regulated in the *UGE* (15-fold) (**Table 3**) and is confirmed by qPCR analysis (47-fold; **Table S11**). Other Aldh isoforms are also increased in either both or one of the primitive populations including *Aldh3b2*, *Aldh1a7* and *Aldh3a1* (**Table 3**) and Aldh activity is increased in APSC (10-fold) (**Figure 3B**). High levels of Aldh are present in hematopoietic and neural SC [46]–[49].

Our promoter analysis supports the notion that retinoid receptors may participate in the transcriptional program of primitive prostate cells, as both the *UGE+Sca-1^Hi^* and the *Sca-1^Hi^-only* clusters are enriched in the signature of the retinoid heterodimer receptor, Rxr (**Tables 2** **,** **S13**). Additionally, the nuclear receptor *Rxra* and two of its binding partners, *Rarb* and *Rarg*, are up-regulated in FPSC (**Table 3**). qPCR analysis confirms elevation of *Rxra* in FPSC (4-fold) and APSC (3-fold), *Rarb* in FPSC (3-fold) and APSC (2-fold) and *Rarg* in APSC (3-fold) (**Table S11**). Thus, a number of the major regulators of the RA/retinoid receptor axis are elevated in FPSC and APSC and their expression in PSC may reflect a ‘tipping-point’ beyond which these cells embark on a differentiation-induced program.

### e) A lipid metabolism signature is evident in FPSC and APSC

Our data indicate that genes related to phospholipid metabolism are up-regulated in the *UGE+Sca-1^Hi^* cluster (**Table S7**), including SC niche-related genes such as *Tiam1* [50] and self-renewal genes such as *Akp2* [51] (**Table 3**). Recent evidence demonstrates the importance of lipids in the self-renewal of SC [52], [53]. Interestingly, the retinoid heterodimer receptor (Fxr/Rxr) complex, whose binding motif is enriched in the promoters of the genes of the *UGE+Sca-1^Hi^* cluster (**Table 2**), is also implicated in the regulation of lipid metabolism [54]. A critical phospholipid transporter, *Mdr3*/*Abcb1a*, whose expression is up-regulated by FXR [55], is also up-regulated in APSC (**Table 3**) as well as several ABC-transporters that are relevant in phospholipid metabolism and that identify SC (**Table 3**). The binding motif of sterol regulatory element-binding factor 1 (Srebp1), an androgen-regulated TF with a role in the metabolism of lipids [56], is enriched in genes of the *UGE+Sca-1^Hi^* and the *Sca-1^Hi^-only* clusters (**Table 2**). Several Srebp1 lipid metabolizing target genes (*Lgals* and *Dlk1*) are up-regulated in FPSC and APSC (**Tables 3** **,** **S13**). Increased expression of *Pltp*, another Srebp1-target gene, in the FPSC (2-fold) and APSC (8-fold) is confirmed by qPCR (**Table S11**). Srebp1 promotes the transcription of HMG-CoA reductase (*Hmgcr*) and fatty acid synthase (*Fasn*). HMG-CoA reductase, an enzyme that catalyzes the conversion of HMG-CoA to mevalonate, a precursor of cholesterol [57], is up-regulated 4-fold, and *Fasn* is up-regulated 2-fold in FPSC (**Table 3**). Interestingly, Srebp1 contributes to the androgen-independent survival and proliferation of prostate cancer cells [58], indicating that lipid metabolism may be relevant to both normal stem and prostate tumor cell biology. In this regard, it is important to note that lipid metabolism in prostate stem cells may not only be relevant for stemness features but may also have an important role in the production of androgens. Prostate tumors have the innate capacity to synthesize their own androgens from cholesterol [59]. In summary, we show that a significant number of factors that regulate lipid homeostasis and are important in SC biology are up-regulated in primitive prostate cells.

### C. Commonalities are evident in the signatures of murine PSC and human prostate cancer

Both prostate carcinoma and benign prostatic hypertrophy are considered to arise from the aberrant proliferation of prostate stem cells. As tumors may express proteins present in their corresponding fetal or primitive adult tissues, an understanding of molecules and pathways expressed by primitive prostate populations may contribute significantly to the development of new therapeutics for proliferative prostatic diseases. A relationship between SC-signatures and cancer signatures has been shown for human hepatocellular carcinoma where the fetal rodent SC signature has prognostic significance for human hepatocellular carcinoma [60]. To determine if genes were expressed in common in murine PSC and human prostate tumors, the signature of murine PSC was compared to the signature of human prostate tumors [61]. For this purpose, gene expression data generated by a screen that compared normal human prostate specimens with primary prostate tumors [61] were used (two-group t-test (*p*<0.05)). This generated two lists of genes: (i) genes that were up-regulated, and (ii) genes that were down-regulated in human prostate tumors. Next, our up-regulated murine PSC profiles were compared with these two lists of genes.

Of the genes expressed in the *UGE-only* cluster, 64 were up-regulated in tumors compared to normal human samples (**Table S15**). Among the PSC-related genes that were up-regulated in tumors are several cell cycle genes (e.g., *Mcm2*, *Cdc6*, and *Pole*). FPSC also express high levels of N-Ras mRNA (4-fold increase), an oncogene that is associated with the development of hormone refractory prostate cancer [62]. Another gene that is up-regulated (2-fold) in FPSC and whose expression is correlated with the progression and aggressiveness of prostate cancer, is *fatty acid synthase* (*Fasn*), an enzyme with a major role lipid metabolism [63]. One of the genes that catalyzes the conversion of testosterone into the more potent androgen, dihydrotestosterone, *Sra5a1*, is up-regulated (8-fold) in the FPSC. This gene may be associated with the progression of prostatic tumors to androgen insensitivity as its transcripts were absent from metastatic prostate lesions but present in primary prostate tumors [64]. Importantly, our PSC profiling also indicates that expression of *Hdac2* is increased in FPSC (**Table 3**). The elevated expression of this enzyme, associated with epigenetic alterations, is correlated with diminished relapse-free survival in prostate cancer [65]. Additionally, numerous chromatin modifiers that promote the cell cycle and prostate tumor progression, including *Ezh2* [66], *Bmi1* [67], *Jarid1b* [68], *Hmga1* [69], *Hmga2* [70], *Dnmt1*, *Dnmt3a* [71] and *Suv39h2* are up-regulated in FPSC (**Table 3**). qPCR analysis confirms increased expression of *Ezh2* (4-fold) in FPSC (**Table S11**). Among the 56 PSC-related genes that are down-regulated in prostate tumors are *Rarb* and *Crabp2*, two mediators of the RA pathway. Decreased expression of these molecules may result in expansion of primitive tumorgenic cells by preventing differentiation. Another PSC-related gene that is down-regulated in prostate tumors is *Smad3*, whose reduced expression may result in proliferation as the inhibitory influence of TGF-β is reduced [33].

**Figure 4:**
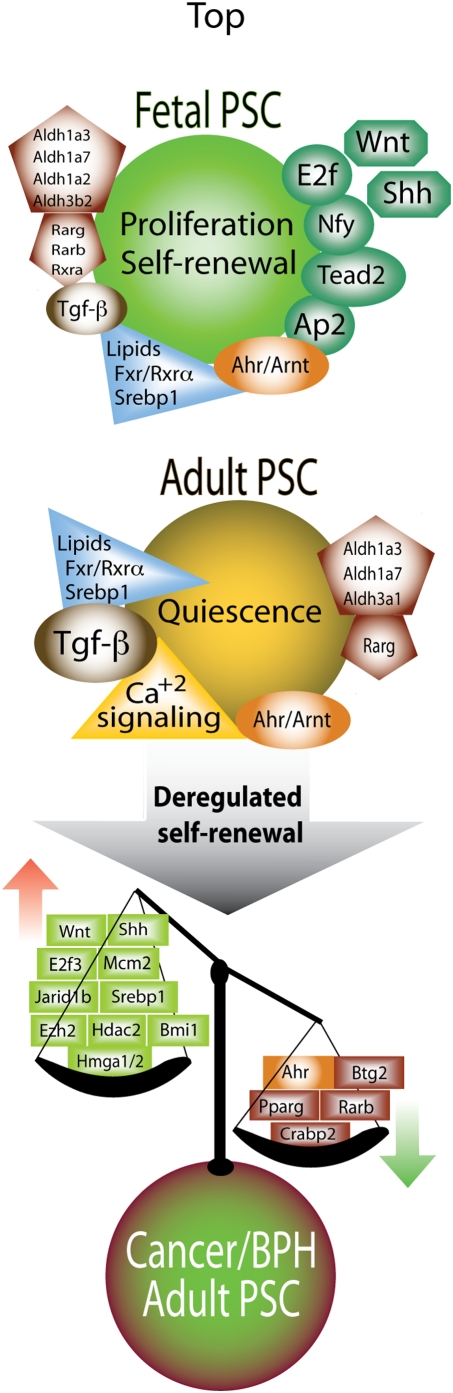
A model of the profiles of fetal PSC and adult PSC indicating commonalities and differences and the manner by which these may alter in proliferative prostatic diseases. Common and distinctive signaling pathways as well as molecules participating in the regulation of self-renewal in fetal PSC and in quiescence in adult PSC were revealed by our gene profiling studies. The unbalanced scales represent possible perturbations in adult PSC that result in the overexpression of certain proto-oncogenes that promote SC self-renewal while other elements that promote differentiation are suppressed, thus leading to deregulated growth. The indicated candidate genes were detected as PSC-related genes that are aberrantly expressed in prostate tumors. Their expression may contribute to the etiology of abnormal PSC proliferation leading to escape from dormancy and unrestrained self-renewal culminating in benign prostate hyperplasia or prostate tumors.

Among the genes present in the *UGE+Sca-1^Hi^* cluster that were down-regulated in prostate tumors (**Table S15**) are the nuclear receptor *Pparg* that mediates lipid metabolism [72] and may act as a tumor suppressor in the prostate [73], and *Parvb* that is decreased in esophageal cancer and may be involved in dedifferentiation and metastasis [74]. Of the genes expressed in the *Sca-1^Hi^-only* cluster, (**Table S15**) *Malat1*, that is associated with endometrial stromal sarcoma, was up-regulated [75]. *Btg2*, which promotes cell quiescence and may be a tumor suppressor in prostate cells [76], was down-regulated. One of the critical events in the development of prostate tumors is gene fusion between the androgen-regulated gene, *TMPRSS2*, and the ETS transcription factor family member *ERG* [77]. Interestingly, we find that expression of both *Erg* (8-fold) and *Tmprss2* (3-fold) was up-regulated in the APSC. The transcription factor Erg is essential for definitive hematopoiesis and the function of normal adult hematopoietic stem cells [78], therefore this molecule has an important role in normal stem cell biology as well as in the promotion of tumorigenesis. Thus, our profiling suggests that the aberrant regulation of certain PSC-related genes may promote the progression of human prostate tumors. In many instances genes that act as tumor suppressors and differentiation inducers were down-regulated, while genes that promote cellular proliferation were elevated in prostate tumors (**Figure 4**).

### Conclusion

In conclusion, based on the complementary findings of functional and transcriptional analyses, the cohort of genes that are active in the FPSC population are largely involved in self-renewal, as would be expected in a proliferating fetal SC population, while adult stem cells have a quiescent signature. The proliferative fetal phenotype may be replicated during the carcinogenic process resulting in the expansion of primitive cells by activating pathways that promote the self-renewal of FPSC. Thus, normally dormant APSC may acquire characteristics of self-renewing primitive fetal prostate cells during oncogenesis (**Figure 4**). Notably, prostate tumors have been shown to originate in the stem cell enriched Sca-1^Hi^ expressing proximal region of ducts [79]. The cohort of stem-related genes that also appear to be deregulated in prostate tumors (**Table S15**) may represent targets for novel therapeutic strategies for treating prostatic diseases. In addition, these molecules may identify a subset of tumors with a more primitive and possibly a more aggressive phenotype. It will be interesting to compare the PSC signature with a large cohort of human prostate tumors as they progress to determine genes that increase or decrease in abundance. These studies may indicate that an alteration in the equilibrium between proto-oncogene and tumor suppressor expression in PSC may favor the evolution of prostate tumors and may also predict their aggressiveness.

## Supporting Information

Figure S1 A two-dimensional principal component analysis mapping of gene expression data from four prostate cell populations. Expression data from the “significant genes”, used in our analysis, were averaged and subjected to principal component analysis (PCA) mapping. The pattern is indicative of good separation of the four populations and reproducibility within the replicate samples in each group. Colors are indicative of the four sample groups.
(3.75 MB TIF)

Table S1 Antibodies used for FACS analyses of SC antigens. Details of all primary antibodies and concentrations used for FACS analysis.
(1.38 MB TIF)

Table S2 Transcripts significantly expressed in stem/progenitor subsets. Lists of transcripts that were significantly highly expressed in each of the subsets (UGE, Sca-1Hi, Sca-1Lo). The relative expression values (LR) compared to the average expression values of control samples (Sca-1Neg) are presented. Each folder contains the data of a separate subset.
(1.07 MB XLS)

Table S3 Transcripts significantly expressed in stem/progenitor clusters. Lists of transcripts that were significantly highly expressed in each of the clusters (UGE-only, UGE+Sca-1Hi, Sca-1Hi-only, Sca-1Hi+Sca-1Lo). The relative expression values (LR) compared to the average expression values of control samples (Sca-1Neg) are presented. Each folder contains the data of a separate cluster.
(0.92 MB XLS)

Table S4 Markers currently described as being expressed in primitive prostate cells and housekeeping genes. The upper half of the table presents transcripts of known prostate stem cell markers that were significantly highly expressed in the stem/progenitor subsets. The lower half of the table presents transcripts of known housekeeping genes and indicates that their expression was not altered between the subsets.
(0.04 MB XLS)

Table S5 Markers currently described as being expressed in SC populations. A list of transcripts previously described as being expressed in stem cell populations that were significantly highly expressed in each of the stem/progenitor-related subsets (UGE, Sca-1Hi, Sca-1Lo). The relative expression values (LR) compared to the average expression values of control samples (Sca-1Neg) are presented.
(0.08 MB XLS)

Table S6 Comparisons of the PSC profile with five other published SC profiles. Lists of transcripts that were significantly highly expressed in each of the clusters (UGE-only, UGE+Sca-1Hi, Sca-1Hi-only, Sca-1Hi+Sca-1Lo) and also known to have elevated expression level in stem cells from other tissues. Each folder presents highly expressed transcripts from a relevant cluster that were also overexpressed in stem cells from another source, as indicated.
(0.74 MB XLS)

Table S7 Gene ontology analysis indicating overrepresented functional categories within gene clusters. Enriched functional categories (P≤0.001, after correction for multiple testing) were identified in each of the stem/progenitor clusters.
(0.48 MB TIF)

Table S8 Functional analysis of stem/progenitor clusters. A list of the genes associated with each of the enriched functional categories that were identified for each of the clusters.
(0.23 MB XLS)

Table S9 Self-renewal genes that are up-regulated in UGE cells. A list of genes whose elevated expression is associated with NSC self-renewal [19] was compared with the list of significantly over-expressed genes in UGE cells. The table represents those genes that were mutually expressed.
(0.10 MB XLS)

Table S10 Wnt pathway and target genes that are up-regulated in UGE cells. The table presents genes that based on the published literature are known to be either participants or targets of the Wnt pathway. Bolded fonts indicate elevated transcript expression.
(0.11 MB XLS)

Table S11 Comparison of gene expression in primitive prostate cell populations as determined by quantitative PCR. qPCR analysis of selected genes from functional categories that were identified in primitive prostate cells. Transcript levels were normalized to the expression of HPRT (housekeeping gene). Data [mean±SD] are presented as the fold change of the expression of each gene in UGE, Sca-1Hi or Sca-1Lo cells relative to its expression in Sca-1Neg cells.
(0.09 MB PDF)

Table S12 Shh pathway and target genes that are up-regulated in UGE and Sca-1Hi cells. The table presents genes that based on the published literature are known to be either participants or targets of the Shh pathway. Bolded fonts indicate elevated transcript expression.
(0.06 MB XLS)

Table S13 Promoter sequence analysis of stem/progenitor clusters. A list of the genes associated with each of the enriched promoter motifs that were identified for the UGE-only, UGE+Sca-1Hi and Sca-1Hi-only clusters.
(0.14 MB XLS)

Table S14 TGF-β target genes in stem/progenitor clusters. A) This list of TGFβ-target genes was obtained after analysis of the supplementary Table 1 of Zavadil et al. (2001) Proc. Natl. Acad. Sci. USA 98 (12), 6686–6691. (10.1073/pnas.111614398). It includes genes that were up-regulated in at least one time point (0.3, 1, 2, 4 hr) after exposure to TGF-β. We compared the list of TGF-β-induced genes (Table A) with our data from three PSC clusters. The genes that are shared are depicted in the following tables: B) UGE-only, C) UGE+Sca-1Hi, D) Sca-1Hi-only.
(0.28 MB XLS)

Table S15 Human prostate cancer signature compared with the murine PSC signature. Gene expression data generated by a screen that compared normal human prostate specimens with primary prostate tumors [60] were used for this analysis. Based on the two-group t-test (p<0.05) we generated two lists of genes that were (i) up- or (ii) down- regulated in human prostate tumors. The up-regulated murine PSC profiles were compared with these two lists of genes. Each folder represents genes from a relevant PSC cluster that were either up- or down- regulated in human prostate tumors
(0.17 MB XLS)
