# Supplementary figures and images for: Molecular Signatures of Prostate Stem Cells Reveal Novel Signaling Pathways and Provide Insights into Prostate Cancer
Source: PLoS One. 2009 May 29;4(5):e5722. doi: 10.1371/journal.pone.0005722 (PMC2684642; doi:10.1371/journal.pone.0005722)

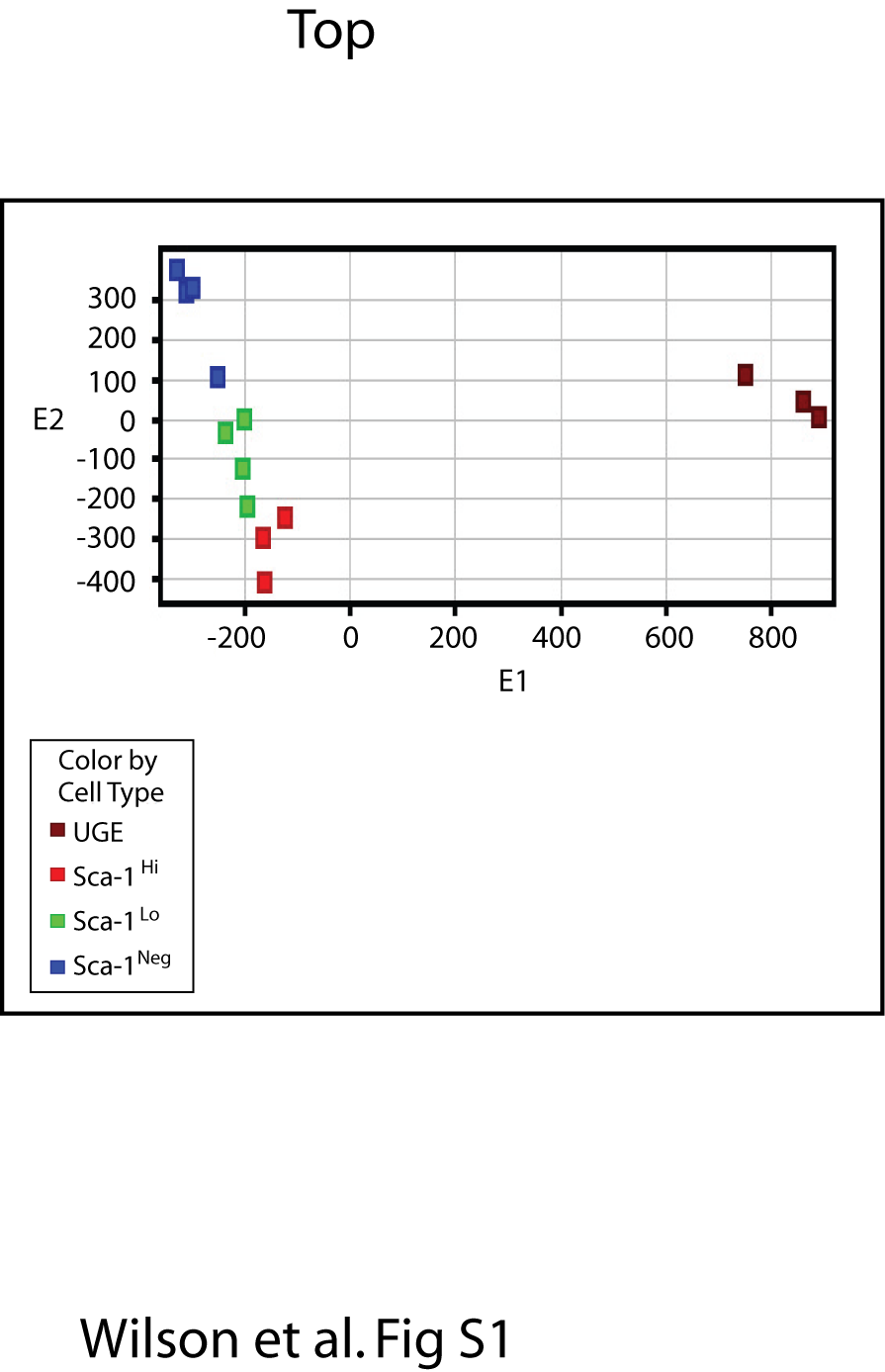

Supplement: Figure S1 — A two-dimensional principal component analysis mapping of gene expression data from four prostate cell populations. Expression data from the “significant genes”, used in our analysis, were averaged and subjected to principal component analysis (PCA) mapping. The pattern is indicative of good separation of the four populations and reproducibility within the replicate samples in each group. Colors are indicative of the four sample groups. (3.75 MB TIF) [file pone.0005722.s001.tif]

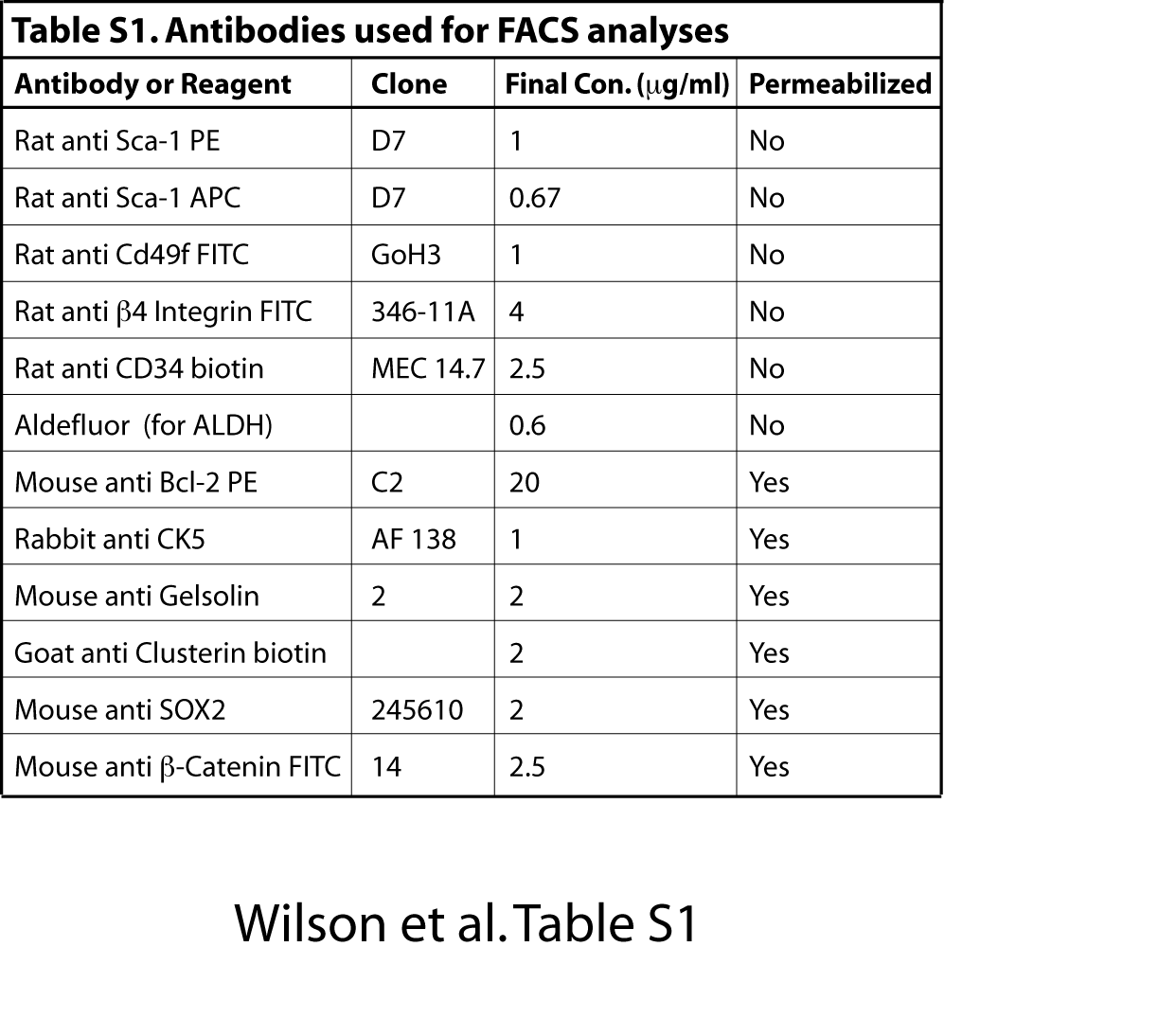

Supplement: Table S1 — Antibodies used for FACS analyses of SC antigens. Details of all primary antibodies and concentrations used for FACS analysis. (1.38 MB TIF) [file pone.0005722.s002.tif]

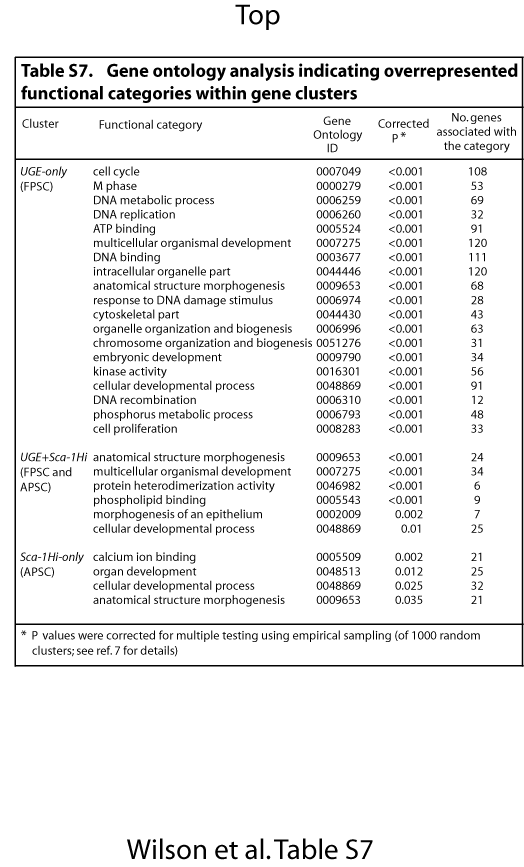

Supplement: Table S7 — Gene ontology analysis indicating overrepresented functional categories within gene clusters. Enriched functional categories (P≤0.001, after correction for multiple testing) were identified in each of the stem/progenitor clusters. (0.48 MB TIF) [file pone.0005722.s008.tif]
