# Supplementary material for: Molecular Signatures of Prostate Stem Cells Reveal Novel Signaling Pathways and Provide Insights into Prostate Cancer
Source: PLoS One. 2009 May 29;4(5):e5722. doi: 10.1371/journal.pone.0005722 (PMC2684642; doi:10.1371/journal.pone.0005722)
Supplement: Table S11 — Comparison of gene expression in primitive prostate cell populations as determined by quantitative PCR. qPCR analysis of selected genes from functional categories that were identified in primitive prostate cells. Transcript levels were normalized to the expression of HPRT (housekeeping gene). Data [mean±SD] are presented as the fold change of the expression of each gene in UGE, Sca-1Hi or Sca-1Lo cells relative to its expression in Sca-1Neg cells. (0.09 MB PDF) [file pone.0005722.s012.pdf]

Top

**Table S11. Comparision of gene expression in primitive prostate cell populations as determined by quantitive PCR**

| Category | Gene | UGE | Sca-1Hi | Sca-1Lo |
|---|---|---|---|---|
| Cell cycle/ Self-renewal | Wnt4 | 5.0 ± 0.4 | 8.4 ± 0.6 | 2.7 ± 0.1 |
| | Wnt6 | 22.4 ± 4.6 | 1.2 ± 0.3 | 1.5 ± 0.6 |
| | Fzd6 | 5.0 ± 0.4 | 3.4 ± 0.2 | 3.0 ± 0.1 |
| | Shh | 35.3 ± 5.9 | 6.3 ± 0.8 | 4.1 ± 1.4 |
| | Gli3 | 3.7 ± 0.2 | 2.9 ± 0.9 | 2.5 ± 0.2 |
| | E2f3 | 3.4 ± 0.2 | 1.3 ± 0.1 | 1.1 ± 0.1 |
| | Cdc2a | 12.1 ± 1.8 | 0.7 ± 0.1 | 1.0 ± 0.5 |
| | Tead2 | 4.1 ± 0.8 | 0.4 ± 0.1 | 0.9 ± 0.3 |
| TGF-β pathway | Clu | 0.1 ± 0.1 | 3.4 ± 0.9 | 2.5 ± 0.2 |
| Ahr pathway | Ahr | 2.4 ± 0.1 | 3.5 ± 0.2 | 1.8 ± 0.2 |
| RA pathway | Crabp2 | 46.6 ± 3.9 | 1.2 ± 0.2 | 2.3 ± 0.4 |
| | Rxra | 4.2 ± 0.4 | 3.3 ± 0.1 | 1.8 ± 0.4 |
| | Rarb | 2.8 ± 0.3 | 1.8 ± 0.1 | 1.3 ± 0.1 |
| | Rarg | 1.5 ± 0.4 | 3.0 ± 0.2 | 2.2 ± 0.1 |
| Phospholipid | Pltp | 1.7 ± 0.3 | 8.1 ± 2.2 | 5.9 ± 1.3 |
| Calcium regulators | Itpr3 | 0.7 ± 0.1 | 1.7 ± 0.3 | 1.1 ± 0.3 |
| | Thbd | 1.5 ± 1.9 | 20.3 ± 6.0 | 10.3 ± 4.5 |
| Chromatin modifiers | Ezh2 | 4.4 ± 0.4 | 1.7 ± 0.1 | 1.3 ± 0.1 |

qPCR analysis of selected genes from functional categories that were identified in primitive prostate cells. Transcript levels were normalized to the expression of HPRT (housekeeping gene). Data [mean ± SD, n=3] are presented as the fold change of the expression of each gene in UGE, Sca-1Hi or Sca-1Lo cells relative to its expression in Sca-1Neg cells.

Wilson et al. Table S11
